# Roles of Neuronal Protein Kinase Cε on Endoplasmic Reticulum Stress and Autophagic Formation in Diabetic Neuropathy

**DOI:** 10.1007/s12035-023-03716-x

**Published:** 2023-10-31

**Authors:** Yu-Yu Kan, Ying-Shuang Chang, Wen-Chieh Liao, Tzu-Ning Chao, Yu-Lin Hsieh

**Affiliations:** 1 School of Medicine, College of Medicine, National Sun Yat-sen University, Kaohsiung, 80424 Taiwan; 2 Department of Anatomy, School of Medicine, College of Medicine, Kaohsiung Medical University, Kaohsiung, 80708 Taiwan; 3 Doctoral Program in Tissue Engineering and Regenerative Medicine, College of Medicine, National Chung Hsing University, Taichung, 40227 Taiwan; 4 Department of Post-Baccalaureate Medicine, College of Medicine, National Chung Hsing University, Taichung, 40227 Taiwan; 5 School of Post-Baccalaureate Medicine, College of Medicine, Kaohsiung Medical University, Kaohsiung, 80708 Taiwan; 6 Department of Medical Research, Kaohsiung Medical University Hospital, Kaohsiung, 80708 Taiwan

**Keywords:** PKCε, ER Stress, Autophagy, IRE1α, Ubiquitin D, PI3Kp85, Diabetic Neuropathy

## Abstract

In chronic diabetic neuropathy (DN), the cellular mechanisms of neuropathic pain remain unclear. Protein kinase C epsilon (PKCε) is an intracellular signaling molecule that mediates chronic pain. This paper addresses the long-term upregulated PKCε in DN associated with endoplasmic reticulum (ER) stress and autophagic formation and correlates to chronic neuropathic pain. We found that thermal hyperalgesia and mechanical allodynia course development were associated with PKCε upregulation after DN but not skin denervation. Pathologically, PKCε upregulation was associated with the expression of inositol-requiring enzyme 1α (IRE1α; ER stress–related molecule) and ubiquitin D (UBD), which are involved in the ubiquitin-proteasome system (UPS)-mediated degradation of misfolded proteins under ER stress. Manders coefficient analyses revealed an approximately 50% colocalized ratio for IRE1α(+):PKCε(+) neurons (0.34–0.48 for M1 and 0.40–0.58 for M2 Manders coefficients). The colocalized coefficients of UBD/PKCε increased (M1: 0.33 ± 0.03 vs. 0.77 ± 0.04, p < 0.001; M2: 0.29 ± 0.05 vs. 0.78 ± 0.04; p < 0.001) in the acute DN stage. In addition, the regulatory subunit p85 of phosphoinositide 3-kinase, which is involved in regulating insulin signaling, exhibited similar expression patterns to those of IRE1α and UBD; for example, it had highly colocalized ratios to PKCε. The ultrastructural examination further confirmed that autophagic formation was associated with PKCε upregulation. Furthermore, PKCεv1-2, a PKCε specific inhibitor, reverses neuropathic pain, ER stress, and autophagic formation in DN. This finding suggests PKCε plays an upstream molecule in DN-associated neuropathic pain and neuropathology and could provide a potential therapeutic target.

## Introduction

Patients with diabetes often experience chronic diabetic neuropathy (DN), which presents as sensory neuropathy symptoms, including pain, tingling, numbness, and skin denervation in the limbs. Skin denervation may induce nerve sensitization [1], which consequently results in chronic pain through the activation of protein kinase C epsilon (PKCε) [2, 3]. PKCε is an important intracellular signaling molecule in primary afferent nociceptors [2, 4, 5]. For example, PKCε upregulation paralleled the skin denervation–associated neuropathic pain [2], suggesting that PKCε in nociceptors has a role in regulating neuropathic pain. However, the function of PKCε has remained controversial because of its protective role in acute stress [6] and its modulatory role in chronic diabetes–related metabolic disorders [2, 7].

Endoplasmic reticulum (ER) stress is induced under hyperglycemia and is also a protective mechanism for eliminating misfolded proteins in DN [8]. There are several enzymes involved in the precise regulation of protein folding and the degradation of misfolded proteins under ER-associated degradation (ERAD) [9, 10] and the ubiquitin-proteasome system (UPS) [11]. The precise regulation requires additional molecular modulation; for example, protein degradation is regulated by PKCε activation, which utilizes UPS in downstream signaling cascades that trigger ubiquitination [12] and the subsequent degradation of PKCε [13, 14]. Moreover, the activation of ubiquitination through the upregulation of ubiquitin D (UBD) further regulates the signaling pathway of inositol-requiring enzyme 1α (IRE1α) under ER stress [15]. The subdomain of ER is involved in phagophore formation, an initial step in autophagy [16]. Pathologically, autophagy is critical in diabetes-associated metabolic diseases [17]. However, the molecular significance of PKCε, ER stress, and autophagic formation in the cellular pathophysiology of nociceptive receptors remains unknown; for example, whether PKCε is activated under hyperglycemia and correlated with the neuropathic pain in DN, which involved ER stress and autophagic formation.

**Fig. 1:**
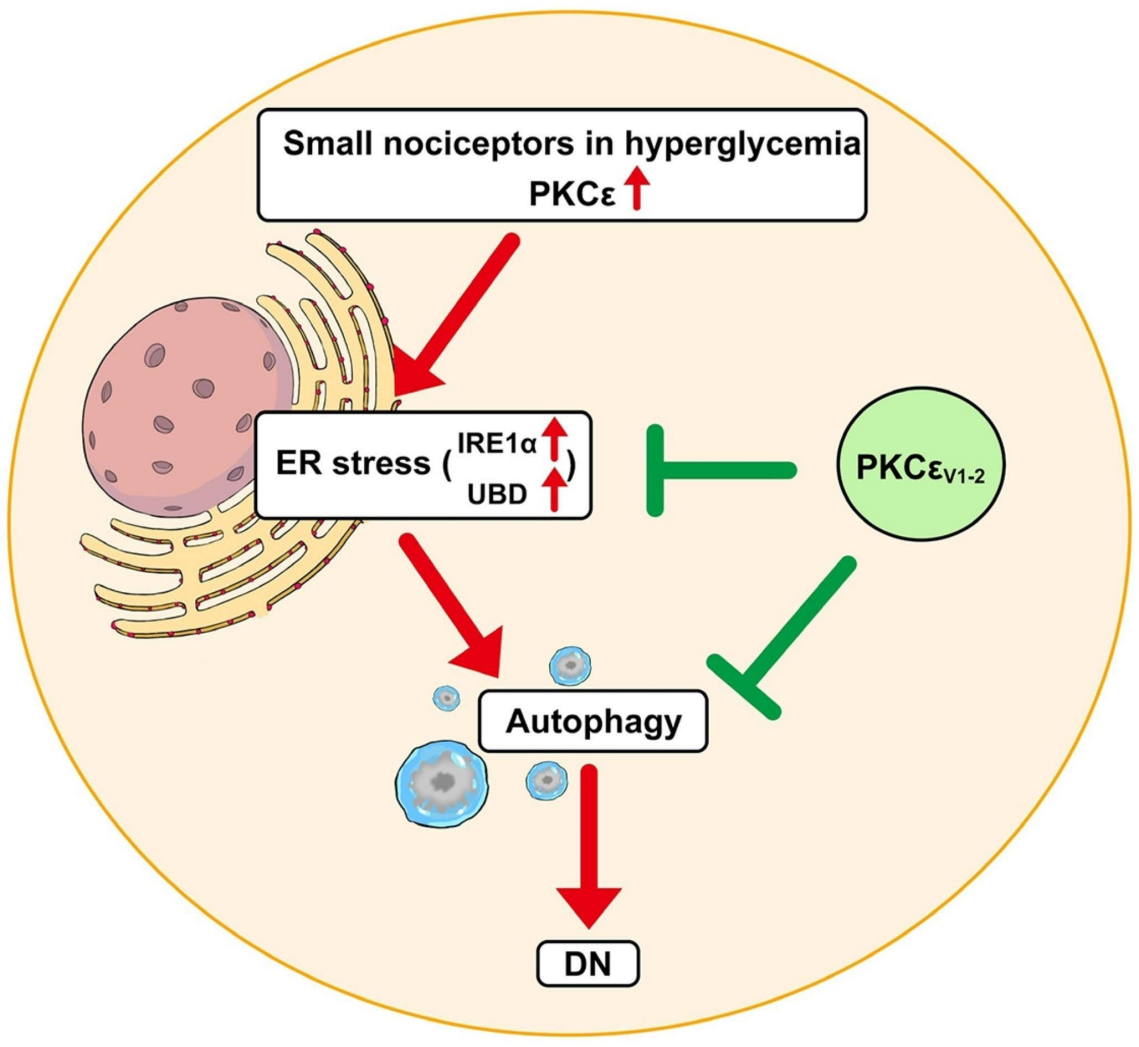
Diagram of regulating endoplasmic reticulum (ER) stress and autophagy by the neuronal ε isoform of protein kinase C (PKCε). PKCε was upregulated under hyperglycemia and subsequentially led to the induction of ER stress and autophagy, which mediated diabetic neuropathy (DN). The pathology of ER stress includes the upregulation of inositol-requiring enzyme 1α (IRE1α) and ubiquitin D (UBD). Upregulated PKCε–mediated ER stress, autophagy, and DN were reversed by PKCεv1-2, a PKCε specific inhibitor

This study investigated the duration of chronic neuropathic pain in DN and examined the coexpression profiles of PKCε with IRE1α, UBD, and p85 of phosphoinositide 3-kinase (PI3Kp85), which are involved in ER stress, dysfunctional protein elimination, and insulin signaling regulation, respectively. The current study also provided ultrastructural evidence of autophagic formation in DN. The inhibition of PKCε with a PKCε-specific inhibitor, namely PKCεv1-2, reversed neuropathic activity, expression of proteins involved in ER stress, and autophagic formation. This finding suggested that PKCε serves as an upstream modulator of ER stress and autophagy in small nociceptors, which mediates DN (Fig. 1).

## Materials and Methods

### Streptozotocin-induced DN and Animal Groups

We established a DN mouse model by administering a single dose of streptozotocin (STZ; 200 mg/kg, Sigma, St. Louis, MO, USA) to 8-week-old C57/B6 mice through intraperitoneal injection. Briefly, their blood glucose levels were examined weekly by using a commercially available glucometer (Accu-Chek Go, Roche Diagnostics GmbH, Mannheim, Germany), and the mice exhibiting hyperglycemia (glucose level > 400 mg/dL) were included in the following experiments (DN group). The mice that exhibited mild-to-moderate hyperglycemia (glucose level < 400 mg/dL) were assigned to the non-DN (nDN) group. The mice that received an equal volume of citrate buffer served as the control (citrate group). Experiments were conducted at different time points on the DN mice in the following subgroups: posttreatment month 1 (DNm1), posttreatment month 2 (DNm2), and posttreatment month 5 (DNm5), and the blood glucose level were measured before sacrificed to ensure the animal in each group met the hyperglycemia for DN (glucose level > 400 mg/dL) and hypoglycemia (glucose level < 400 mg/dL) for nDN criteria. To eliminate the bias of age factors, the citrate group included each corresponding time of experimental tissues. The mice were housed in plastic cages under a 12-h light–dark cycle with *ad libitum* access to food and water. Without glycemic control, the survival of DN mice was limited after 5 months of STZ administration. Therefore, the endpoint of this study was set at DNm5, and all procedures were coded and blinded and minimized animal suffering by ethical guidelines.

### Neuropathic Pain Evaluation

The activity and appearance of the mice were checked before the evaluation, which consisted of tests of thermal (hot plate test) and mechanical (von Frey monofilament test) responses.

*Hot Plate test*. The mice were placed on a 52 °C hot plate (IITC, Woodland Hills, CA, USA) enclosed in a Plexiglas cage. The withdrawal latency of the hindpaw to thermal stimulation was determined to an accuracy of 0.1 s. Each test session comprised three trials at 30-min intervals, and the withdrawal criteria included shaking, licking, or jumping from the hot plate. The mean latency was expressed as the threshold of each animal to thermal stimulation.

*von Frey Monofilament test*. The changes in the mechanical threshold of each group were assessed using the up-and-down method with different calibers of von Frey monofilaments (Somedic Sales AB, Hörby, Sweden) in accordance with our established protocol [18]. In brief, a series of monofilaments were applied to the plantar region of the hindpaw. If paw withdrawal occurred, a monofilament of a smaller caliber was applied; however, if the paw was not withdrawn, a monofilament of a larger caliber was applied. Four additional stimuli with monofilaments of various calibers were applied on the basis of the preceding responses, and the mechanical thresholds were calculated using a formula [8].

### Evaluation and Quantitation of Protein Gene Product 9.5(+) Intraepidermal Nerve Fibers

We revealed intraepidermal nerve fibers (IENFs) by using a pan-axonal marker, protein gene product (PGP)9.5, in immunohistochemical studies. Briefly, the mice received intracardiac perfusion with 0.1 M phosphate buffer (PB), followed by 4% paraformaldehyde in 0.1 M PB. After perfusion, the footpad skin was postfixed for another 6 h and stored in PB. For cryosection, the footpad skin was cryoprotected with 30% sucrose in PB overnight and sectioned perpendicular to the epidermis (30-µm thickness) on an HM440E sliding microtome (Microm, Walldorf, Germany). Footpad sections were incubated with anti-PGP 9.5 (1:1000, UltraClone, Isle of Wight, UK) antiserum following a regular immunostaining protocol, and the reaction product was visualized using 3,3ʹ-diaminobenzidine (Sigma). For quantitation, PGP9.5(+) IENFs were counted, and the quantified criteria were conducted following established criteria in a coded manner [8, 19]. IENF density was defined as the number of IENFs divided by the epidermal length (fibers/mm).

### Investigation and Quantification of Dorsal Root Ganglion Neurons with Different Phenotypes

We assessed the pathological profiles of the fourth- and fifth-lumbar dorsal root ganglion neurons (DRG; L4 and L5, respectively) through immunofluorescence. These DRGs were cryoprotected with 30% sucrose in PB overnight, and 8-µm-thick cryosections were obtained using a cryostat (CM1850, Leica, Wetzlar, Germany). For adequate sampling, two ganglia per mouse and 5–8 sections per DRG tissue (at 80-µm intervals) were immunostained. The primary antisera were anti-phosphorylated PKCε (p-PKCε; goat, 1:200; Santa Cruz Biotechnology, Santa Cruz, CA, USA), anti-UBD (rabbit, 1:200; Proteintech, Rosemont, IL, USA), anti-IRE1α (rabbit, 1:600; Abcam, Cambridge, MA, USA), and anti-PI3Kp85 (rabbit, 1:500, Merck Millipore, Darmstadt, Germany). We employed the following combinations of primary antisera: (1) IRE1α:p-PKCε, (2) UBD:p-PKCε, and (3) PI3Kp85:p-PKCε. Briefly, after overnight incubation with primary antisera, the sections were incubated with Texas Red (TR; 1:100, Jackson ImmunoResearch, West Grove, PA, USA) and fluorescein isothiocyanate (FITC)-conjugated secondary antisera (1:100, Jackson ImmunoResearch) corresponding to the appropriate primary antisera for 1 h. For DRG quantification, each DRG section was systematically photographed at 200× under a fluorescence microscope (Axiophot microscope, Carl Zeiss, Oberkochen, Germany) with appropriate filters. A montage of the entire DRG section was obtained according to our established protocols [8, 18]. To identify labeled neurons, optical intensities between immunoreactive and background neurons were determined. For the FITC signal, the optical intensities were set between 130 and 245 on a 0–255 scale by using an FITC filter. For the TR signal, the thresholds were set in the range of 125–250 by using a TR filter. Each signal of a fluorochrome below these ranges served as the background. To avoid bias in neuronal density measurements, only neurons with a clear nuclear profile were counted. The neuronal areas were measured using ImageJ version 1.44d.

### Ultrastructural Examination of Autophagy

Ultrastructural examination of autophagy was conducted using DRG tissues. Briefly, DRG tissues were postfixed with 5% glutaraldehyde in 0.1 M PB overnight and further fixed in 2% osmium tetraoxide for 2 h. Tissues were then dehydrated through a graded ethanol series and embedded in Epon 812 resin (Polyscience, Philadelphia, PA, USA). Thin sections (50 nm) were stained with uranyl acetate and lead citrate and were then observed and photographed using an electron microscope (Hitachi, Tokyo, Japan).

### Pharmacological Intervention: PKCε Inhibitor Administration

To examine the role of PKCε in DN-related neuropathology and neuropathic pain, PKCε activity was inhibited using a pharmacological, PKCε-specific inhibitor, PKCεv1-2 (Calbiochem, La Jolla, CA, USA; Stock: 2 mg/ml). Briefly, the drugs were freshly prepared with normal saline and delivered through a lumbar puncture (1 µg/5 µL) [20] using a Hamilton microsyringe (Hamilton, Reno, NV, USA) [18]. To investigate the pharmacological effects of the PKCεv1-2 inhibitor, two administration protocols were performed: (1) on the next day after STZ treatment, administration every 2 days for 4 weeks (DNw4; mPKCεI group; cumulative dose: 15 µg/mouse) and (2) administration of normal saline by using the same protocol to serve as a negative control (vePKCεI group). After treatment, the mice were housed in plastic cages under a 12-h light–dark cycle with *ad libitum* access to water and food. Changes in neuropathic pain were assessed at week 1 (DNw1), week 2 (DNw2), week 3 (DNw3), and DNw4 after PKCεv1-2 inhibitor administration.

### Statistical Analysis

To minimize individual variation, each group had 5–8 animals. All data are expressed as the mean ± standard derivation of the mean. One-way repeated-measures analysis of variance (ANOVA) followed by Tukey’s *post hoc* test was performed, and p < 0.05 was considered statistically significant.

## Results

### STZ-induced Chronic Neuropathic pain was Independent of Denervation of IENFs

**Fig. 2:**
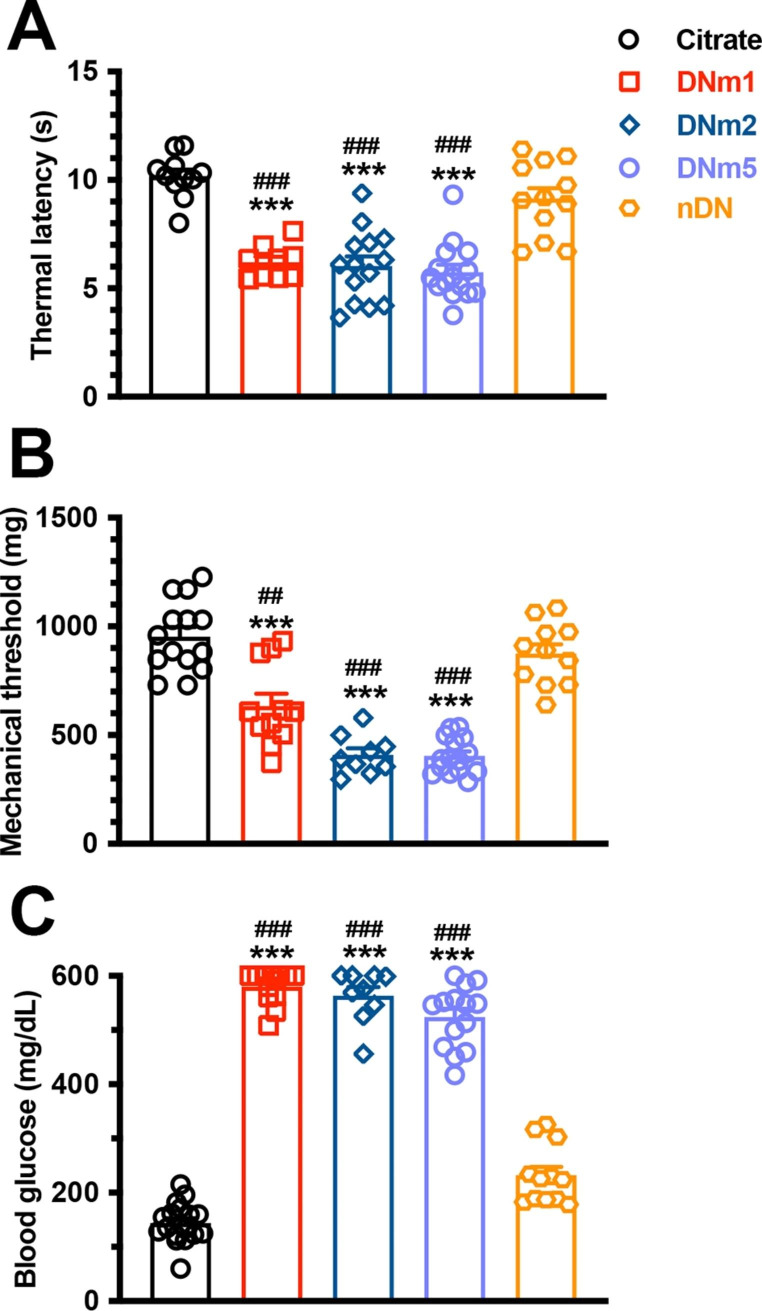
Development of neuropathic pain in streptozotocin (STZ)-induced diabetic neuropathy (DN). A mouse model of DN was generated through intraperitoneal injection of STZ (200 mg/kg). Neuropathic pain were evaluated through the hot plate test (**A**) and von Frey hair test (**B**), and levels of blood glucose were measured in the citrate, DNm1 (blood glucose > 400 mg/dL at posttreatment month 1), DNm2 (blood glucose > 400 mg/dL at posttreatment month 2), DNm5 (blood glucose > 400 mg/dL at posttreatment month 5), and nDN (blood glucose < 400 mg/dL) groups. Group labels are indicated on each graph. (**A**–**C**) Scatter plot and bar graphs show the changes in thermal latencies (**A**), mechanical thresholds (**B**), and levels of blood glucose (**C**). Thermal hyperalgesia and mechanical allodynia were observed in DNm1, DNm2, and DNm5 mice. ***p < 0.001: DNm1, DNm2, or DNm5 group versus citrate group. ##p < 0.01, ###p < 0.001: DNm1, DNm2, or DNm5 group versus nDN group

**Fig. 3:**
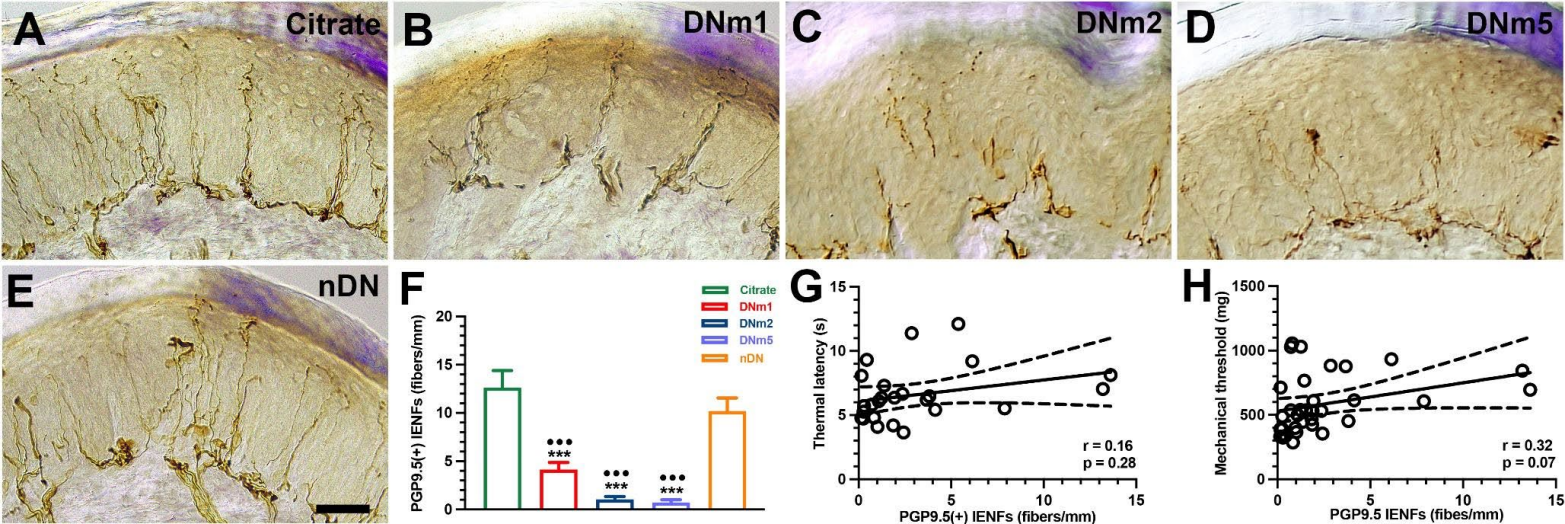
Reduction of intraepidermal nerve fibers (IENFs) in streptozotocin (STZ)-induced diabetic neuropathy (DN). (**A**–**E**) IENFs visualized using the pan-axonal marker, protein gene product (PGP)9.5. PGP9.5(+) IENFs arose from the subepidermal nerve plexus with a typical varicose appearance in the citrate group (**A**). The profiles of PGP9.5(+) IENFs were markedly reduced in the DNm1 (blood glucose > 400 mg/dL at posttreatment month 1; **B**), DNm2 (blood glucose > 400 mg/dL at posttreatment month 2; **C**), and DNm5 (blood glucose > 400 mg/dL at posttreatment month 5; **D**) groups, but not in the citrate (**A**) and nDN (blood glucose < 400 mg/dL; **E**) groups. Bar, 50 μm. (**F**) Bar graphs show the quantitative results of PGP9.5(+) IENF density in the above groups. (**G**, **H**) Correlations of PGP9.5(+) IENF densities with thermal latencies (**G**) and mechanical thresholds (**H**)

The DNm1 mice exhibited both thermal hyperalgesia (6.0 ± 0.6 vs. 10.3 ± 0.6 s, p < 0.001) and mechanical allodynia (603.1 ± 168.8 vs. 931.9 ± 147.2 mg, p < 0.001), which were comparable with those in the citrate and nDN groups (thermal sensation: 9.4 ± 1.6 s, p < 0.001; mechanical thresholds: 888.7 ± 143.6 mg, p < 0.001). These neuropathic pain were also observed in the DNm2 (thermal latencies: 6.0 ± 1.7 s, p < 0.001; mechanical thresholds: 409.1 ± 89.4 mg, p < 0.001) and DNm5 mice (thermal latencies: 5.7 ± 1.3 s, p < 0.001; mechanical thresholds: 404.4 ± 82.1 mg, p < 0.001; Fig. 2A and B). Blood glucose examinations revealed hyperglycemia in the DNm1, DNm2, and DNm5 groups, suggesting that the blood glucose level was associated with neuropathic pain (Fig. 2C). Skin pathology also revealed persistent skin denervation; for example, the density of PGP9.5(+) IENFs was progressively reduced in the DNm1 (3.6 ± 1.6 fibers/mm, p < 0.001), DNm2 (1.0 ± 0.8 fibers/mm, p < 0.001), and DNm5 (0.7 ± 0.7 fibers/mm, p < 0.001) mice compared with those in the citrate (12.7 ± 3.5 fibers/mm) and nDN mice (11.2 ± 3.6 fibers/mm; Fig. 3A-F). However, no correlation was observed between the PGP9.5(+) IENF density and thermal latency (r = 0.16, p = 0.28; Fig. 3G) or mechanical threshold (r = 0.32, p = 0.07; Fig. 3H).

### Coexpression of PKCε(+):IRE1α(+) and PKCε(+):UBD(+) Neurons was Correlated with Neuropathic Pain after STZ-induced DN

**Fig. 4:**
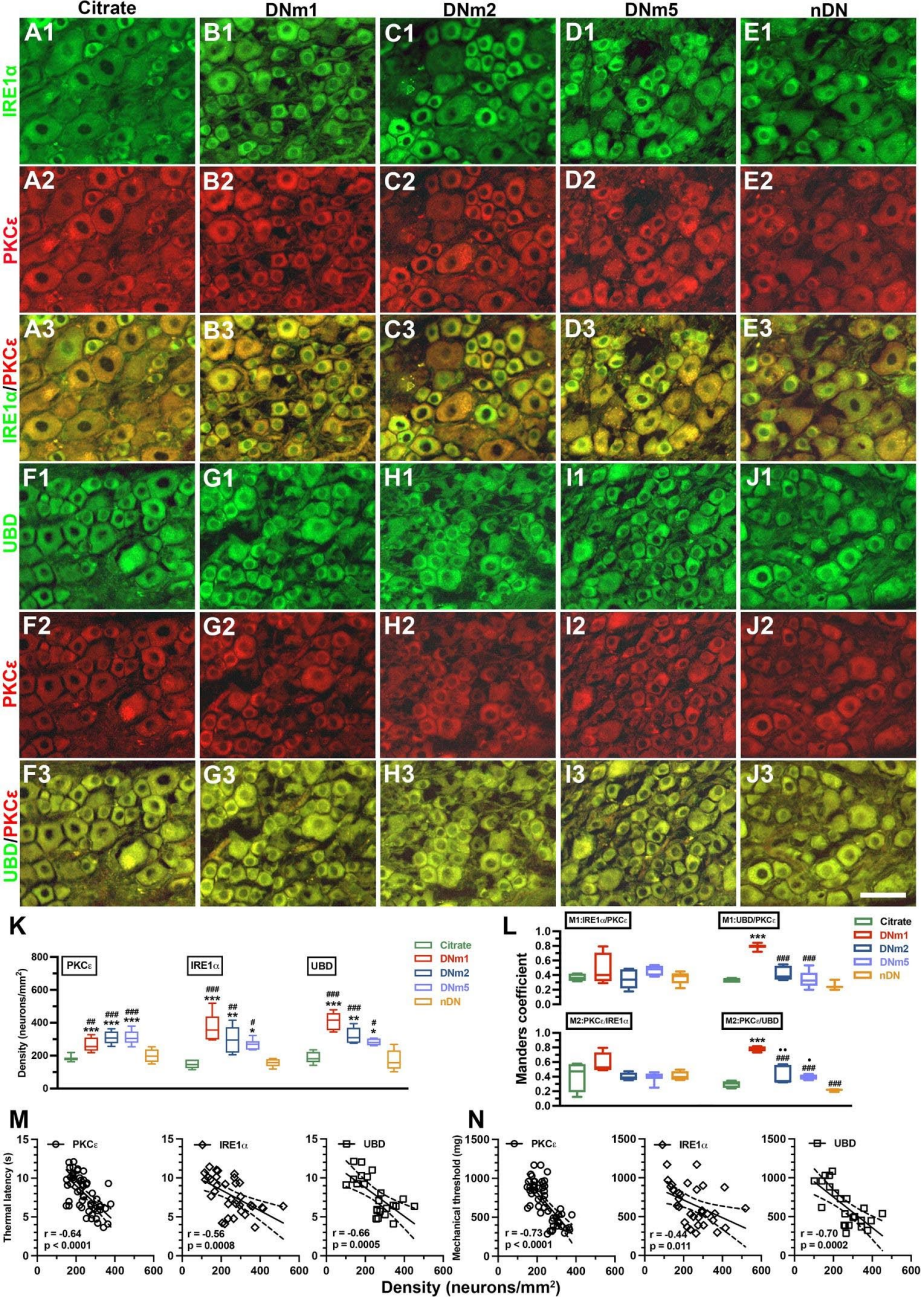
Colocalization of neuronal ε isoform of protein kinase C (PKCε) and endoplasmic reticulum (ER) stress–related molecules of inositol-requiring enzyme 1α (IRE1α) or with ubiquitin D (UBD)(+) neurons in streptozotocin (STZ)-induced DN. (**A**–**J**) Double-labeling immunofluorescence staining was performed in two combinations—(1) IRE1α (**A**1–**E**1, green) with PKCε (A2–E2, red) and (2) UBD (**F**1–**J**1, green) with PKCε (**F**2–**J**2, red)—in the dorsal root ganglion of the citrate (**A**1–**A**3 and **F**1–**F**3), DNm1 (blood glucose > 400 mg/dL at posttreatment month 1; **B**1–**B**3 and **G**1–**G**3), DNm2 (blood glucose > 400 mg/dL at posttreatment month 2; **C**1–**C**3 and **H**1–**H**3), DNm5 (blood glucose > 400 mg/dL at posttreatment month 5; **D**1–**D**3 and **I**1–**I**3), and nDN (blood glucose < 400 mg/dL; **E**1–**E**3 and **J**1–**J**3) groups. Photographs of IRE1α(+) and PKCε(+) neurons (**A**3–**E**3) as well as UBD(+) and PKCε(+) neurons (**F**3–**J**3) are merged for analyzing the colocalization pattern. Bar, 50 μm. (**K**) PKCε(+), IRE1α(+), and UBD(+) neurons are increased in the DNm1, DNm2, and DNm5 mice. (**L**) M1 (upper panel) and M2 Manders coefficients (lower panel) of IRE1α(+) and PKCε(+) (right panel) and UBD(+) and PKCε(+) neurons (left panel) according to colocalized patterns in Fig. 4**A**3–4**E**3 and 4**F**3–4**J**3. Group labels are indicated on each graph. *p < 0.05, **p < 0.01, ***p < 0.001: DNm1, DNm2, or DNm5 group versus citrate group. ##p < 0.01, ###p < 0.001: DNm1, DNm2, or DNm5 group versus nDN group. •p < 0.05, ••p < 0.01: DNm2 or DNm5 group versus DNm1 group. (**M, N**) Densities of PKCε(+) (open circles), IRE1α (+) (open diamonds), and UBD(+) (open squares) neurons are inverse to the changes of thermal latency (**M**) and mechanical threshold (**N**)

Because the activation of ERAD and UPS might contribute to the development of neuropathic pain, we first performed immunostaining of DRG sections for investigating PKCε and IRE1α expression. IRE1α (Fig. 4A1–4E1) and PKCε (Fig. 4A2–4E2) were preferentially expressed by small-diameter neurons. Notably, both IRE1α(+) (372.9 ± 83.1 vs. 149.1 ± 26.8 neurons/mm^2^, p < 0.001) and PKCε(+) neurons (264.4 ± 42.1 vs. 185.5 ± 16.0 neurons/mm^2^, p < 0.001) were upregulated in the DNm1, DNm2 (IRE1α: 294.4 ± 78.0 neurons/mm^2^, p = 0.002; PKCε: 309.0 ± 37.5 neurons/mm^2^, p < 0.001), and DNm5 (IRE1α: 268.5 ± 32.3 neurons/mm^2^, p = 0.016; PKCε: 309.0 ± 42.3 neurons/mm^2^, p < 0.001) mice, but not in the citrate and nDN mice (IRE1α: 156.1 ± 24.1 neurons/mm^2^, p < 0.001; PKCε: 197.5 ± 37.9 neurons/mm^2^, p = 0.001; Fig. 4A1–4E3 and Fig. 4K). The Manders coefficients revealed that the expression of M1 IRE1α/PKCε (0.34–0.48) and M2 PKCε/IRE1α (0.4–0.58) was similar, indicating approximately 50% colocalization of IRE1α:PKCε neurons (Fig. 4L). We then examined the expression of UBD, the upstream modulator in ubiquitination. UBD(+) neurons exhibited similar neuronal upregulation patterns (Fig. 4F1–4J1); for example, UBD(+) neurons were upregulated in the DNm1 (384.6 ± 59.0 vs. 189.3 ± 35.5 neurons/mm^2^, p < 0.001), DNm2 (320.7 ± 49.6 neurons/mm^2^, p = 0.003), and DNm5 mice (282.4 ± 20.8 neurons/mm^2^, p = 0.03; Fig. 4K). The M1 Manders coefficients of UBD/PKCε (0.77 ± 0.04 vs. 0.33 ± 0.03; p < 0.001) and the M2 Manders coefficients of PKCε/UBD in the DNm1 mice (0.78 ± 0.04 vs. 0.29 ± 0.05; p < 0.001) were higher than those in the citrate group, suggesting that coexpression of UBD:PKCε neurons may increase the risk of acute DN (Fig. 4L). Furthermore, linear regression revealed that PKCε(+), IRE1α(+), and UBD(+) neuronal densities were inversely correlated with thermal latency (Fig. 4M) and the mechanical threshold (Fig. 4N).

### Coexpression of PKCε(+):PI3Kp85(+) Neurons was Correlated with Neuropathic Pain in DN

**Fig. 5:**
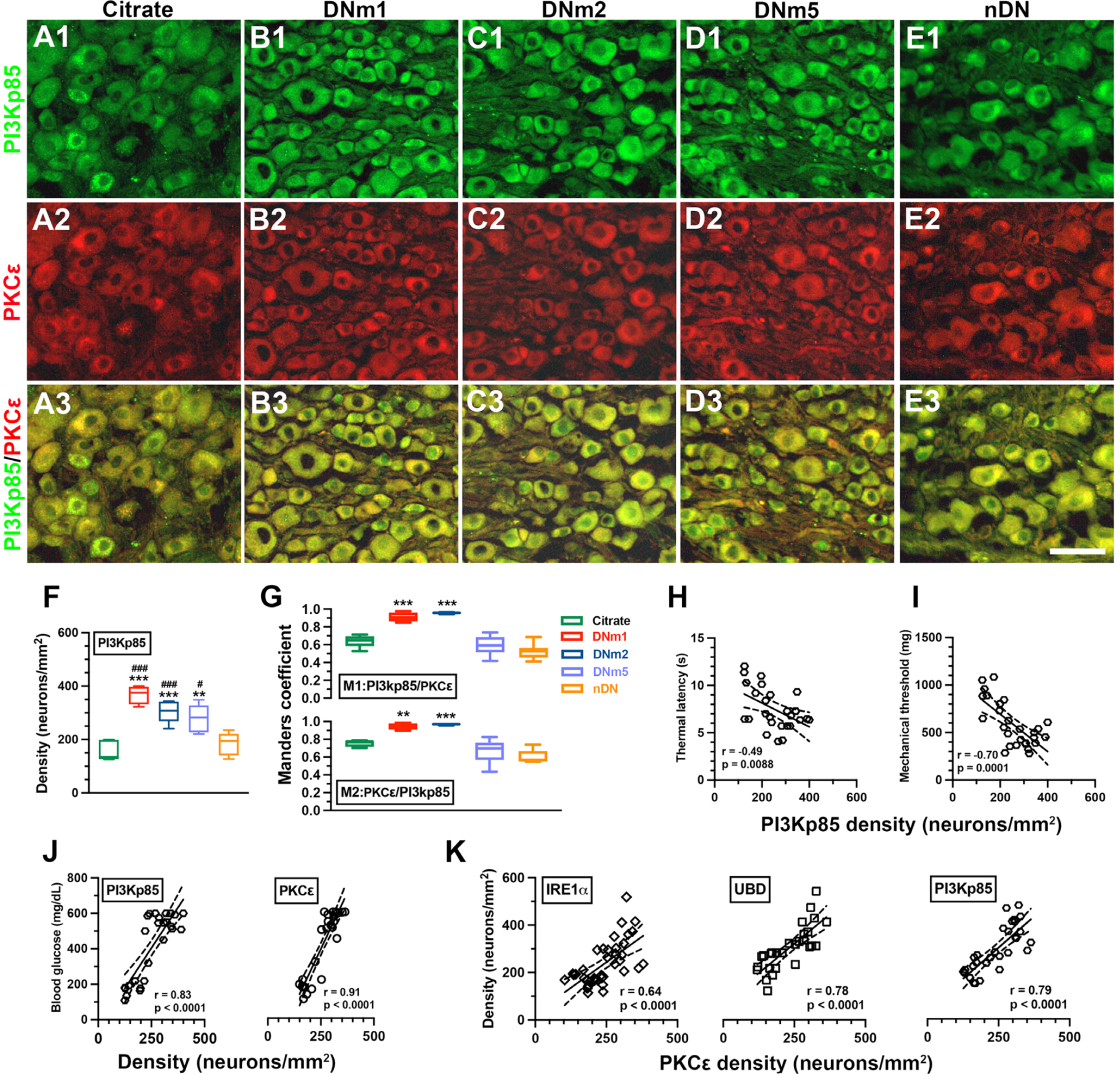
Colocalization patterns of the regulatory subunit p85 of phosphoinositide 3-kinase (PI3Kp85) and neuronal ε isoform of protein kinase C (PKCε) in dorsal root ganglia (DRG) neurons after streptozotocin (STZ)-inducted DN. Double-labeling immunofluorescence staining was performed with anti-PI3Kp85 (**A**1–**E**1, green) and anti-PKCε (**A**2–**E**2, red) in the DRG of the citrate (**A**1–**A**3), DNm1 (blood glucose > 400 mg/dL at posttreatment month 1; B1–B3), DNm2 (blood glucose > 400 mg/dL at posttreatment month 2; **C**1–**C**3), DNm5 (blood glucose > 400 mg/dL at posttreatment month 5; **D**1–**D**3), and nDN (blood glucose < 400 mg/dL; **E**1–**E**3) groups. Photographs of PI3Kp85(+) and PKCε(+) neurons (**A**3–**E**3) are merged to analyze the colocalization pattern. Bar, 50 μm. (**F**, **G**) Box graphs show the density of PI3Kp85(+) neurons (**F**) and M1 (**G**, upper panel) and M2 Manders coefficients (**G**, lower panel) of PI3Kp85(+) and PKCε(+) neurons according to the colocalization patterns in A3–E3. Manders coefficients indicate that the upregulation of PI3Kp85 and PKCε were synchronized in DNm1 and DNm2 mice. Group labels are indicated on each graph. **p < 0.01, ***p < 0.001: DNm1, DNm2, or DNm5 group versus citrate group. #p < 0.05, ###p < 0.001: DNm1, DNm2, or DNm5 group versus nDN group. (**H**, **I**) PI3Kp85(+) neuronal densities are inversely correlated to the thermal latency (H) and mechanical threshold (I). (**J**) PI3Kp85(+) (left panel) and (right panel) PKCε(+) neuronal densities are linearly correlated to the levels of blood glucose. (**K**) The density profiles of IRE1α (open diamonds, left panel), UBD (open squares, middle panel), and PI3Kp85 (open circle, right panel) show linear correlation to PKCε

Given that PI3Kp85 is a regulatory subunit of PI3K that suppresses insulin signaling, we investigated the change in the coexpression of PI3Kp85 and PKCε (Fig. 5). We found that PI3Kp85 was also expressed by small-diameter neurons and was upregulated in the DNm1 (368.3 ± 34.9 vs. 156.1 ± 38.2 neurons/mm^2^, p < 0.001), DNm2 (301.3 ± 38.2 neurons/mm^2^, p < 0.001), and DNm5 (278.4 ± 52.9 neurons/mm^2^, p = 0.001) mice, but not in the citrate and nDN mice (Fig. 5A1–5E1 and Fig. 5F). The M1 Manders coefficients of PI3Kp85/PKCε (0.91 ± 0.05 vs. 0.64 ± 0.07, p < 0.001) and the M2 Manders coefficients of PKCε/PI3Kp85 (0.94 ± 0.04 vs. 0.75 ± 0.04, p = 0.007) in the DNm1 and DNm2 mice (M1: 0.96 ± 0.01, p < 0.001; M2: 0.97 ± 0.01, p < 0.001) were higher than those in the citrate mice (Fig. 5G). PI3Kp85(+) neuronal density was also inversely correlated with thermal latency (r = − 0.49, p = 0.0088; Fig. 5H) and mechanical threshold (r = − 0.70, p = 0.0001; Fig. 5I). PI3Kp85(+) neuronal density exhibited a linear relationship with blood glucose levels (r = 0.83, p < 0.0001), and this linear correlation was also found in PKCε (r = 0.91, p < 0.0001; Fig. 5J). Furthermore, PKCε(+) neuronal density was also linear to IRE1α (r = 0.64, p < 0.0001), UBD (r = 0.78, p < 0.0001), and PI3Kp85 (r = 0.79, p < 0.0001) (Fig. 5K). Accordingly, The PKCε expression patterns were correlated to ER stress, misfolded protein responses, and hyperglycemia.

### PKCε Inhibition Reversed Neuropathic pain, ER Stress and Misfold Protein–related Pathology

**Fig. 6:**
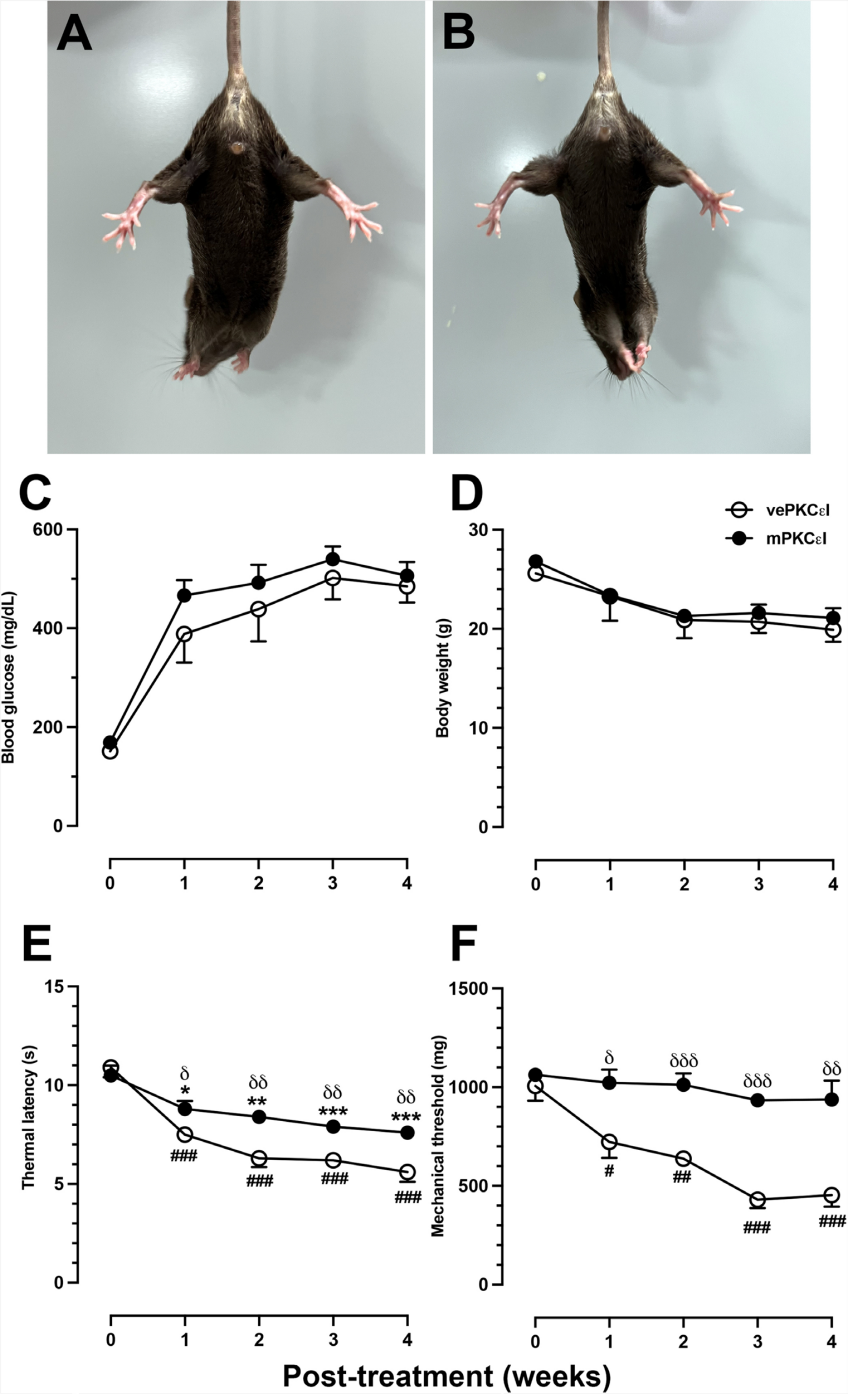
PKCε inhibitor, PKCεv1-2, reversed neuropathic pain but had no effect on relieving hyperglycemia in streptozotocin (STZ)-induced diabetic neuropathy (DN). PKCεv1-2 (cumulative dose: 15 µg/mouse) was administered through lumbar punctures, as described in Materials and Methods section. (**A, B**) The gross appearance of vePKCεI (**A**) and mPKCεI (**B**) mice. Mice had a normal hindlimb extension in both groups. vePKCεI, DN mice received multiple doses of vehicle; mPKCεI, DN mice received multiple doses of PKCεv1-2. (**C, D**) The graphs show the changes of blood glucose (**C**) and (**D**) body weight in vePKCεI (opened circle) and mPKCεI (filled circle) mice. (**E, F**) Neuropathic pain were evaluated using the hot plate test (**E**) and von Frey hair test (**F**) in vePKCεI (opened circle) and mPKCεI (filled circle) mice. mPKCεI mice had relief of thermal hyperalgesia and mechanical allodynia compared to vePKCεI mice. *p < 0.05, **p < 0.01, ***p < 0.001: posttreatment vs. pretreatment of mPKCεI mice. #p < 0.05, ##p < 0.01, ###p < 0.001: posttreatment vs. pretreatment of vePKCεI mice. δp < 0.05, δδp < 0.01, δδδp < 0.001: vePKCεI vs. mPKCεI group

**Fig. 7:**
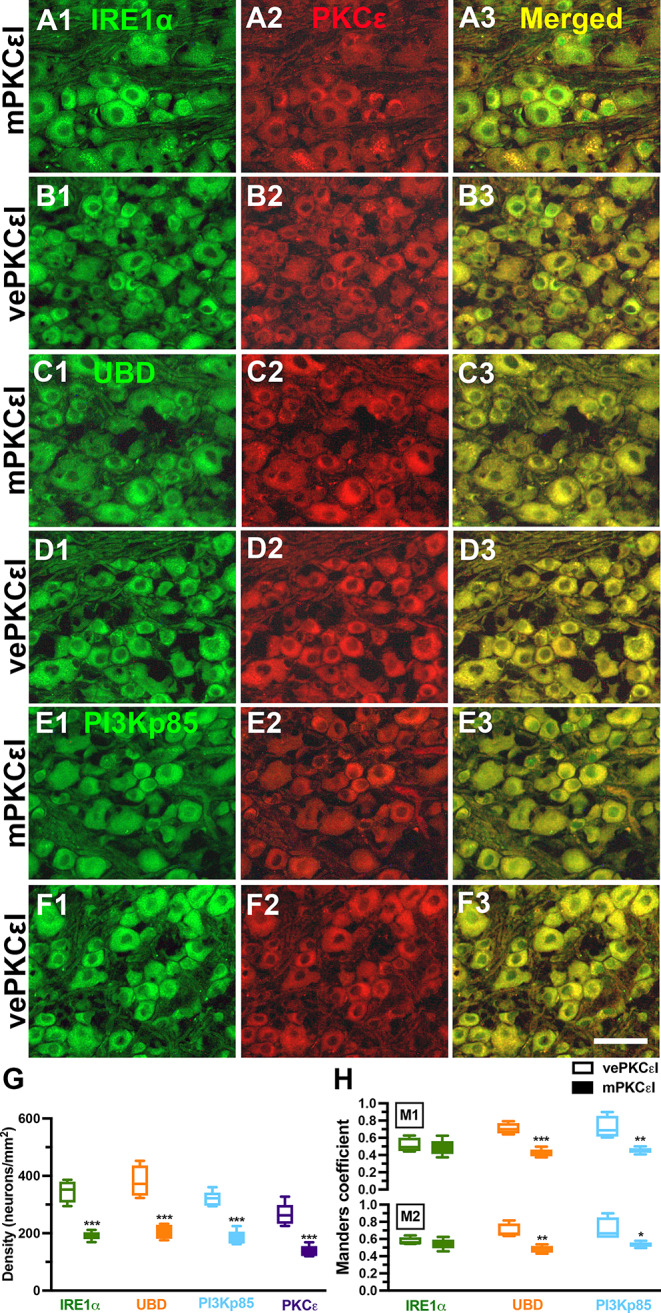
PKCε inhibitor, PKCεv1-2, administration downregulated neuronal ε isoform of protein kinase C (PKCε) and endoplasmic reticulum (ER) stress–related molecules in streptozotocin (STZ)-induced DN. (**A**–**F**) Double-labeling immunofluorescence staining was performed in three combinations—(1) inositol-requiring enzyme 1α (IRE1α; **A**1, **A**3, and **B**1, **B**3 in green), (2) ubiquitin D (UBD; **C**1, **C**3 and **D**1, **D**3 in green), and (3) regulatory subunit p85 of phosphoinositide 3-kinase (PI3Kp85; **E**1, **E**3 and **F**1, **F**3 in green) with PKCε (**A**2–**F**2 and **A**3–**F**3, in red)—in the dorsal root ganglion in mice receiving multiple doses of PKCεv1-2 inhibitor (mPKCεI; **A**, **C** and **E**) or vehicle (vePKCεI; **B**, **D** and **F**). Photographs of IRE1α(+) and PKCε(+) neurons (**A**3, **B**3), UBD(+) and PKCε(+) neurons (**C**3 and **D**3), as well as PI3Kp85(+) and PKCε(+) neurons (**E**3 and **F**3) are merged for analyzing the colocalization pattern. Bar, 50 μm. (**G, H**) The graph shows (**G**) the density changes of IRE1α(+), UBD(+), PI3Kp85(+), and PKCε(+) neurons in mPKCεI (filled color box) and vePKCεI (opened color box) and (**H**) M1 (upper panel) and M2 Manders coefficients (lower panel) of corresponding neurons according to colocalized patterns in Figs. **A**3–**F**3. IRE1α(+), UBD(+), PI3Kp85(+), and PKCε(+) neurons are decreased in mPKCεI mice. Group labels are indicated on each graph. *p < 0.05, **p < 0.01, ***p < 0.001: mPKCεI vs. vePKCεI group

To test the regulation roles of PKCε in neuropathic pain, ER stress and misfolded protein–related pathology, we applied PKCε pharmacological blockade through a lumbar puncture. Both the mPKCεI and vePKCεI mice exhibited normal gait and extension responses on the hind limb after repeated administration of the PKCε inhibitor, indicating the absence of damage to the lumbosacral plexus (Fig. 6A vs. 6B), and PKCεI also not affected on hyperglycemia (Fig. 6C) or body weight (Fig. 6D). In neuropathic pain assessment, the vePKCεI mice exhibited thermal hyperalgesia from DNw1 (7.5 ± 0.3 vs. 10.9 ± 1.1 s, p = 0.002) to DNw4 (5.6 ± 1.1 s, p < 0.001). Although the mPKCεI mice also developed thermal hyperalgesia at DNw1 (8.8 ± 0.9 vs. 10.5 ± 1.1 s, p = 0.021), thermal latencies were higher in the mPKCεI mice than in the vePKCεI mice from DNw1 (8.8 ± 0.9 vs. 7.5 ± 0.3 s, p = 0.03) to DNw4 (7.6 ± 0.6 vs. 5.6 ± 1.1 s, p = 0.007; Fig. 6E). By contrast, there was no decrease of mechanical thresholds—similar to baseline values—was observed in the mPKCεI mice from DNw1 (1022.5 ± 147.7 vs. 722.9 ± 162.1 mg, p = 0.02) to DNw4 (937.8 ± 214.1 vs. 453.9 ± 118.8 mg, p = 0.005) compared with those in the vePKCεI mice (Fig. 6F). The expression profiles of PKCε and ER stress–related molecules were correlated with pharmacological PKCε blockade. For example, the downregulation profiles of IRE1α (Fig. 7A1 and 7A3), UBD (Fig. 7C1 and 7C3), and PI3Kp85 (Fig. 7E1 and 7E3) were in parallel to the expression profiles of PKCε in the mPKCεI mice which compared with the vePKCεI mice (Fig. 7G). Colocalization analyses also demonstrated similar patterns of these molecules in relation to pathological manifestations; lower M1 Manders coefficients of UBD/PKCε (0.42 ± 0.05 vs. 0.71 ± 0.06, p < 0.001) and PI3Kp85/PKCε (0.45 ± 0.03 vs. 0.72 ± 0.13, p = 0.003) were observed in the mPKCεI mice compared with those in the vePKCεI mice (Fig. 7H, upper panel). The M2 Manders coefficients were similar to the M1 Manders coefficients (Fig. 7H, lower panel).

### Autophagic Formation in STZ-induced DN and PKCε Inhibition Reversed Autophagic Formation

**Fig. 8:**
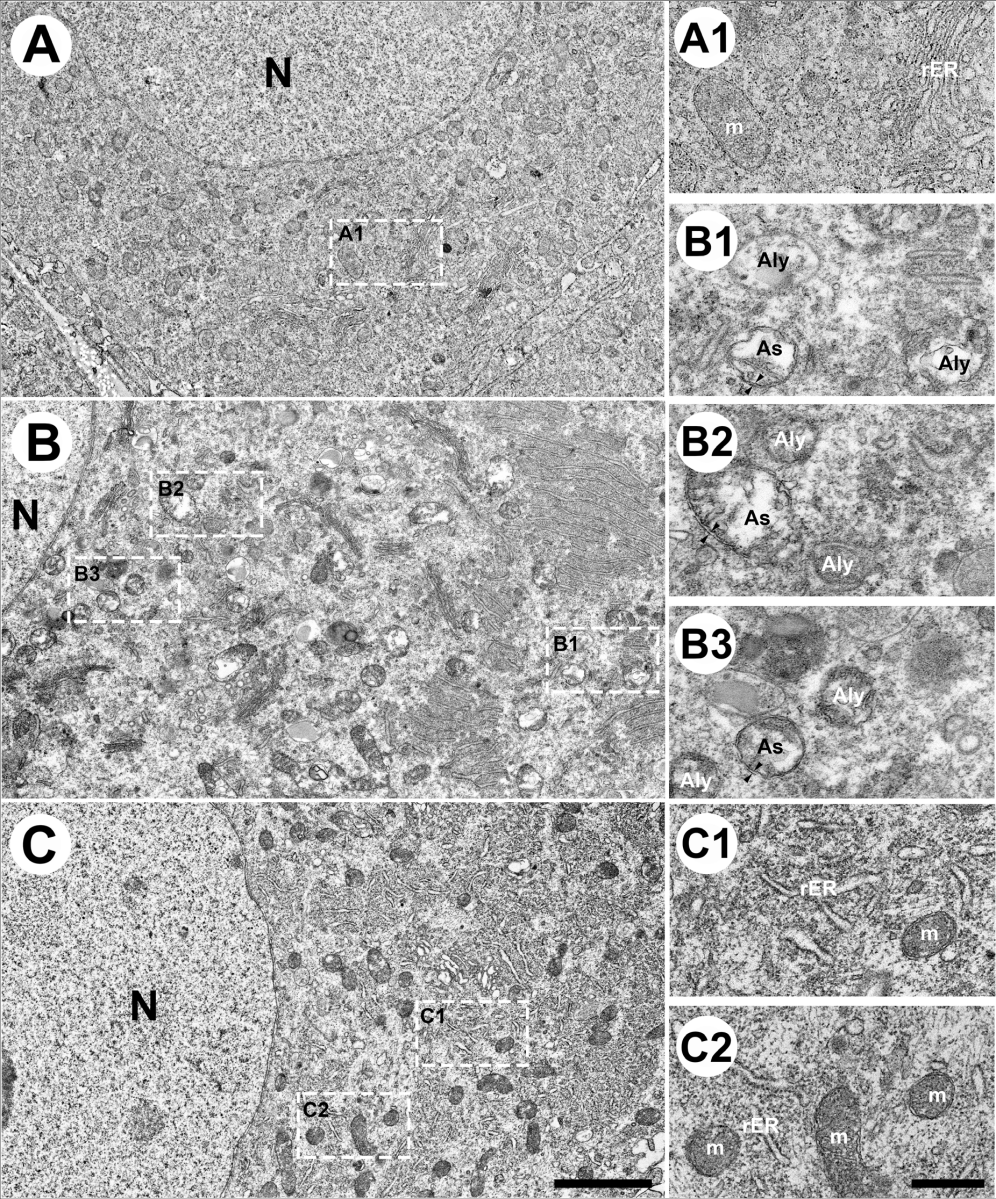
Ultrastructural examination of autophagy formation in streptozotocin (STZ)-induced DN. (**A**–**C**) The lumbar dorsal root ganglia of the citrate (**A**), DN (**B**), and (**C**) DN mice that received the PKCεv1-2 inhibitor (mPKCεI) with samples prepared for electron microscopy examinations. Ultrastructural examinations of the rough endoplasmic reticulum (rER) system (5000×) next to the cell nucleus (N; **A**–**C**), exhibiting flattened stacks of cisternae (ER system) and abundant mitochondria (m). Bar, 1 μm. (**A**1–**C**2) Higher magnification (15,000×) in the insets of Figure **A**–**C**. (**A**1) rER in the citrate mice appeared as a flattened membrane-bound cisternae with a lucent lumen. The double membranous structure of mitochondria was also observed. By contrast, the DN mice (**B**1–**B**3) had numerous vacuoles that were filled with amorphous or granular substances referred to as an autophagosome (As), the early autophagic vacuoles, and autolysosome (Aly), the late autophagic vacuoles. Some autophagosomes had a double-limiting membrane (arrowheads in **B**1–**B**3). (**C**1, **C**2) mPKCεI mice exhibited flattened membrane-bound cisternae of rER and normal mitochondria appearance. Bar, 250 nm

Since ER stress initiates autophagic formation [14], we further performed ultrastructural examinations of DRGs to confirm whether PKCε blockade could reverse the autophagic formation in DN (Fig. 8). The rough ER (rER) comprised stacks of flattened membrane-bound cisternae with a lucent lumen. Double-membrane mitochondria were observed in the citrate group (Fig. 8A and inset in 8A1). By contrast, numerous swollen vesicles represented the degradation of rER and the mitochondria (Fig. 8B), which were designated as the early autophagosome (As in Fig. 8B1–8B3) and late autolysosome stages of autophagy (Aly in Fig. 8B1–8B3) in the DN mice. The amorphous masses in the swollen vesicles implied the accumulation of misfolded or unfolded proteins; additionally, we observed some autophagosomes with a double-limiting membrane [21] within the DRG soma (As in Fig. 8B1–8B3). In contrast, the mPKCεI mice exhibited stacks of flattened membrane-bound cisternae and normal mitochondrial appearance, similar to the citrate mice (Fig. 8C, 8C1, and 8C2), indicating the inhibition of PKCε activity reversed autophagic formation.

## Discussion

This study demonstrated that progressive chronic neuropathic pain in DN parallels PKCε expression but not IENF density. This study further highlights PKCε is an upstream molecule that regulates the expression of (1) IRE1α, an ER stress–related molecule, (2) UBD, a regulator of misfolded protein degradation and (3) PI3Kp85, an insulin signaling molecule; all these molecules correlate with the levels of neuropathic pain in DN, as demonstrated by pharmacological inhibition of PKCε. In addition, this study also revealed that ER stress and autophagic formation in the DRG of DN were alleviated by pharmacological PKCε blockade.

### PKCε for Evaluating DN-related Neuropathic Pain

IENF density assessed through PGP 9.5 immunohistochemistry using skin biopsy is currently a reliable clinical diagnostic method for predicting the progression of small-fiber neuropathy [22]. However, some patients with diabetes with relatively few IENFs experience no pain in quantitative sensory testing [23], and our previous report demonstrated that diabetic mice with low IENF density had no neuropathic pain [2, 8]. These findings suggest that additional potential biomarkers in the DRG neurons are required for evaluating neuropathic pain in DN. PKCε has been demonstrated to be an intracellular signaling messenger for long-lasting hyperalgesic priming and chronic pain [3, 5]. However, the pathophysiology of neuropathic pain in DN differs from hyperalgesic priming caused by peripheral local damage. The pathology of DN involves both central sensitization and peripheral IENFs degeneration; in particular, central sensitization leads to the development of neuropathic pain, as has been demonstrated in our previous studies [8, 19]. Our previous study also demonstrated that PKCε upregulated on injured nociceptors correlated to developing neuropathic pain in DN [2]. Accordingly, this report suggested PKCε plays a master signal response correlated to neuropathic pain after IENF degeneration, as demonstrated by this current report, such as PKCε inhibition alleviated the neuropathic symptoms.

### PKCε Activation and ER Stress Response for Neuropathic Pain

It is important to note that challenges, such as diabetic hyperglycemia, can impair the ER intrinsic pathway for protein folding and quality control, accumulating protein aggregates. ER stress and autophagy functions are similar, namely, to eliminate unfolded and misfolded proteins [17, 24–26]. Therefore, they maintain the physiological homeostasis of the intracellular environment. Notably, ER stress and autophagy occur in several metabolic disorders, particularly neurological disorders [27–29]. ER stress eliminates misfolded proteins by triggering an adaptive signaling cascade that is required to activate the ERAD [9, 10] and UPS [11] systems. The current study found that PKCε was upregulated in small nociceptors under hyperglycemia and was highly colocalized with IRE1α and UBD; these molecules are involved in the ERAD and UPS systems, respectively. The expression of PKCε also linear to IRE1α, UBD, and PI3Kp85, suggesting that PKCε modulates ER stress through several downstream signaling cascades.

Although the pathology of ER stress could be diminished by targeting a specific ER stress signal pathway via various inhibitors, they still exhibit limited physiological effects, such as having a poor effect in alleviating neuropathic pain in DN [30]. Accordingly, targeting upstream modulators of ER stress is an additional therapeutic approach [31]. In the current study, the PKCε inhibitor reversed the ER stress pathology and neuropathic pain behaviors without affecting blood glucose levels. These findings suggest that PKCε is an upstream modulator of ER stress, autophagy, and neuropathic pain. Therefore, the inactivation of PKCε might be a potential therapeutic approach.

### PKCε Activation Mediates Autophagy

Pathologically, autophagy plays a housekeeping role by removing misfolded proteins and eliminating intracellular pathogens, suggesting that it is a critical process in the pathogenesis of human diseases such as DM-associated metabolic diseases [17]. Previous reports have suggested that the downregulation of the PKCε signal is associated with decreased autophagic formation, which is also demonstrated by the increased anti-apoptotic effect for cancer cell lines [32, 33]. However, the role of autophagy in DN is ambiguous [34, 35]; for example, an ultrastructural study demonstrated that autophagic adaptation was enhanced in the peripheral nerve fibers of patients with diabetes [36]. However, other studies have observed reduced autophagic pathology in diabetic animals [35, 37]. Although previous studies have focused on exploring autophagic pathology, upstream modulation remains unknown. This report demonstrated that PKCε upregulation was parallel to autophagic pathology, as phagophores and autophagosomes were observed in the DN mice. PKCε inhibition reversed autophagy formation and neuropathic pain manifestations. Based on our findings, there seems to be an association between the formation of autophagy and the development of neuropathic pain.

Additionally, PKCε may be an upstream modulator for ER stress and autophagy. Notably, this report does not address the causal relationship between ER stress, autophagic pathology, and the development of neuropathic pain in DN. Therefore, this issue needs further investigation. Overall, this study is the first report to provide comprehensive evidence of molecular and pathological roles of PKCε, ER stress, autophagic formation, and insulin signaling in DRG neurons. The study suggests that PKCε is an effective therapeutic target in DN.

## Data Availability

The data that support the findings of this study are available on request from the corresponding author upon reasonable request.

## Abbreviations

N: Nucleus
m: Mitochondria
rER: Rough endoplasmic reticulum
Aly: Autolysosome
As: Autophagosome
